# Neonatal Lupus presenting with neonatal hemochromatosis-like liver disease that responded to steroids: a case report

**DOI:** 10.1186/s12887-022-03713-4

**Published:** 2022-11-03

**Authors:** Ammar Abdulaziz Khayat, Amani Jaboor Alkhaldi

**Affiliations:** 1 Dpeartment of Pediatrics, College of Medicine, Umm Al Qura University, Al Abdeyah, Alawali, Makkah, 24381 Saudi Arabia; 2 Pediatric Gastroenterology Unit, King Abdulaziz University Hospital, Jeddah, Saudi Arabia

**Keywords:** Neonatal lupus erythematosus, Neonatal cholestasis, Neonatal hemochromatosis, Gestational alloimmune liver disease, Anti-smooth muscle antibodies, Sjogren Syndrome type A antibodies, Sjogren Syndrome type B antibodies, Case report

## Abstract

**Background:**

Neonatal lupus erythematosus is a rare multisystem autoimmune disorder that predominantly involves the heart with congenital heart block but can involve other organs including the liver. The disease results from passage of maternal autoantibodies to the fetus and manifests in various forms depending on the organ involved. Neonatal lupus liver disease manifestations range from benign elevation in aminotransferases to fatal hepatic insufficiency with iron deposition that does not respond to therapy. Only a handful of cases have been reported to date. The antibodies implicated are Sjogren Syndrome types A and B antibodies. Other non-specific autoantibodies can be positive as well such as antinuclear antibodies. Smooth muscle antibodies are classically considered specific to autoimmune hepatitis, and while they have been described in other chronic liver diseases, they have not been described in neonatal lupus liver disease. Herein we report a rare case of neonatal cholestasis due to neonatal lupus liver disease that presented with a positive smooth muscle antibodies in addition to a biochemical picture of neonatal hemochromatosis, with a remarkably elevated ferritin, that responded well to steroid therapy.

**Case presentation:**

An 8-day old full-term baby girl was referred to our center for evaluation of neonatal bradycardia and generalized jaundice that started in the first day of life. Prenatal history was significant for fetal bradycardia. Examination was unremarkable except for bradycardia and generalized jaundice. Laboratory findings included elevated alanine aminotransferase, aspartate aminotransferase, Alkaline Phosphatase, and total and direct bilirubin. Her ferritin was markedly elevated along with triglycerides. Sjogren syndrome antibodies were positive in addition to antinuclear and anti-smooth muscle antibodies. The diagnosis of cardiac neonatal lupus was given, and her liver disease was attributed to lupus despite the biochemical picture of neonatal hemochromatosis. She was started on oral prednisolone for which her liver function parameters showed a dramatic response and continued to be within the normal limits several weeks after discontinuation of steroids.

**Conclusion:**

Neonatal lupus liver disease is a rare cause of neonatal cholestasis that can rarely present with neonatal hemochromatosis picture which unlike other causes of neonatal hemochromatosis can be reversed with steroid therapy.

## Background

Neonatal lupus erythematosus (NLE) is a multisystem autoimmune disorder that results from passage of maternal autoantibodies to the fetus in utero, and manifests based on the system or systems being involved. [1–3]The most common manifestations involve the heart with congenital heart block, followed by cutaneous, hematologic, and hepatobiliary manifestations, with the central nervous system being rarely involved as well. [1–3] Liver involvement had been described in some case reports and is estimated to be around 10% of all NLE cases based on a national registry in the United States. [4] The real prevalence from a multinational prospective study may be as high as 24%. [5] Outside of those studies, literature on this association is limited to case reports and it appears to be rare still. Liver involvement may occur with or without other systems’ involvement. [6–9] The range of manifestations could be as mild as transient elevations in aminotransferases, or more significant with cholestasis or even neonatal acute liver failure (NALF) with neonatal hemochromatosis-like picture. [2, 4, 10–12]

The diagnosis is usually made when the NLE specific autoantibodies are detected in the baby along with evidence of liver affection, in the appropriate clinical context when other etiologies are reasonably excluded such as biliary atresia. [2, 4, 13] Those autoantibodies are mainly Sjogren Syndrome type A (SSA/anti La) and Sjogren Syndrome type B (SSB/anti Ro) antibodies that have been described in the early 1990s in association with an unexplained neonatal hepatitis and neonatal hemochromatosis (NH) cases and were later found to be of maternal origin. [8, 14] Other autoimmune markers that are associated with various autoimmune liver diseases include anti-nuclear antibodies (ANA), smooth muscle antibodies (SMA), both of which are parts of the diagnostic criteria for autoimmune hepatitis type 1 (AIH-1), liver kidney microsomal antibodies that are highly specific to AIH type – 2, anti-mitochondrial antibodies, and anti-Saccharomyces Cerevisiae antibodies (ASCA) that are found in primary biliary cholangitis, and primary sclerosing cholangitis. [15–17] They are not specific to autoimmune liver diseases nor do have a well-known pathogenic role apart from liver kidney microsomal antibodies. [18] They have been described in various other liver disorders including non-alcoholic fatty liver disease, and viral hepatitides, among others. [19]

Herein we describe a case of NLE associated cholestatic liver disease that presented with a positive SMA, in addition to a biochemical picture of neonatal hemochromatosis, with a remarkably elevated ferritin, that -unlike other fatal cases- had a complete resolution of the liver injury with steroid therapy.

### Case presentation

An 8-day-old full-term girl baby was referred to our center for evaluation and management of fetal and neonatal bradycardia. Our gastroenterology service was consulted due to generalized jaundice that started on her first day of life. She had no history of fever, vomiting, diarrhea, respiratory issues, or abnormal movements. There was no history of change in the color of her urine. Her stools were well pigmented. There was no history of phototherapy use. Perinatal history revealed a term infant, born to a healthy 40-year-old mother, gravida 3 Para 2, with no maternal history of infections, cholestasis, rheumatological, autoimmune disorders, or medications use during pregnancy. She was delivered via low segment cesarian section due to fetal bradycardia. Her prenatal ultrasound images were all unremarkable. Her birth weight was 2700 g. APGAR scores were 8, and 9, at 5, and 10 min respectively. She received positive pressure ventilation shortly after birth, and her heart rate was in the 60’s so she was electively intubated and mechanically ventilated at a community hospital prior to her transfer to our center on day of life 8. There was no similar history in her other siblings nor where there any history of fetal loss, or cholestasis in any family member. The baby was born to un-consanguineous parents. Examination was significant for bradycardia with heart rates in the 60 s while on therapy with isoproterenol (b adrenergic), generalized icterus, and a palpable liver 2 cm below the costal margin. Otherwise, her examination was unremarkable with stable vitals, growth parameters on the 25^th^ centile for age, negative dysmorphism, normal tone and central nervous system examination, and no visible cutaneous legions. Workup revealed a transient thrombocytopenia at 62 K/uL (reference range 150–450) that resolved on subsequent samples, otherwise a normal complete blood count, chemistry panel, urea nitrogen, and creatinine. Her liver panel revealed a cholestatic picture with total protein at 49 g\L(reference range 57–82), albumin at 29.5 g\L (reference range 40.2–47.6), alkaline phosphatase (ALP) at 173 U\L (reference range 46–116), gamma glutamyl transferase (GGT) at 61 U\L (reference range for adult males < 73 and adult females < 38), alanine amino transferase (ALT) at 44 U\L (reference range 10–49), aspartate amino transferase (AST) at 42 U\L (reference range 34–118), total bilirubin 331 at umol\L (reference range 5–21) (19 mg/dl), and a direct bilirubin at 245 umol\L (reference range 0–5) (14 mg/dl), and her coagulation profile was unremarkable with the highest international normalized ratio being 1.38 that responded to vitamin K therapy. Additional laboratory workup including serologies for toxoplasma, herpes simplex virus, cytomegalovirus, syphilis (TORCH), human immunodeficiency virus, blood and urine cultures, thyroid function panel, and newborn screen, were unremarkable. Since gestational alloimmune liver disease (GALD) was in the differential; Ferritin was sent and came back high at 2772 ng/ml reference range13-150) along with triglycerides at 3.42 mmol\L (Normal < 1.7 mmol/L). Imaging including cardiac echo and liver ultrasound were unremarkable with a normal liver size and echogenicity, a normal common bile duct size, and a normal gallbladder with no sludge seen. Electrocardiography confirmed the presence of a third-degree atrioventricular block. Due to this history, NLE was highly suspected so autoimmune markers were sent that showed a positive SS-A/Ro antibodies, ANA at a titer of 1:640 and a speckled pattern, and SMA at a titer of 1:20, whereas Double stranded deoxyribonucleic acid antibodies, Smith extractable nuclear antigen (Sm-ENA) antibodies, SS-B/La antibodies, AMA, LKM, Parietal cell antibodies were all negative. Immunoglobulin levels were all within normal levels. Based on the autoantibody profile and the cardiac findings the patient was diagnosed with congenital heart block due to cardiac- NLE, as well as cholestasis due to NLE-liver disease. Our cardiac team placed a transvenous pacemaker, and we started the patient on oral prednisolone at a dose of 1 mg/kg for 6 weeks. While the patient’s heart condition was stable on temporary pacing and b adrenergics, her liver parameters showed a significant improvement only after steroids were introduced. Her liver parameters were all normalized within 4 weeks and continued to be within the normal ranges 2 weeks after steroids were tapered off as shown in Table 1. Her most recent liver parameters 8 weeks after discontinuation of steroids are all within normal limits as well. Her mother was later screened for lupus, for which she had a positive ANA, and rheumatoid factor.

## Discussion and conclusions

The case described herein follows the usual presentation of cardiac NLE, and NLE-liver disease.

Cardiac NLE is estimated to be around 15–30% of all NLE cases with the most common manifestation being complete (3.^rd^ degree) heart block. [20] Liver disease in NLE may occur with or without cardiac involvement. [2, 4, 5] The most common described manifestation is elevated levels of aminotransferases that follow a benign course and resolves spontaneously. [4, 5] Other manifestations including cholestasis (specifically conjugated hyperbilirubinemia), NALF, and hepatomegaly detected by imaging have been described with decreasing frequency as well. [4, 9, 11, 12] The case herein falls in line with the described cholestasis and hepatomegaly, although the GGT was not remarkably elevated early in the disease course, a finding classically found in progressive familial intrahepatic cholestasis types 1 and 2 as well as bile acid synthesis disorders. [21]

The pathophysiology of NLE-liver disease is not fully understood. Much like autoantibodies in AIH-1, it is not known whether SSA/Ro and SSB/La autoantibodies have a direct pathogenic role in liver injury associated with NLE. [22] They seem to target specific serotonergic receptors in the conduction system of the heart, and similar antigens appear to be expressed in different tissues including the liver. [22] Earlier reports of NLE-liver disease postulated that the liver injury is secondary to the hemodynamic compromise that results from congenital heart block. [11] But more recent reports described a hepatitis picture distinct from congestive hepatopathy based on the histological changes seen in liver biopsies of NLE-liver disease patients, including non-specific giant cell hepatitis, portal inflammation, and extramedullary hematopoiesis among others. [11, 23] An interesting histologic finding that had been rarely described in fatal cases of NLE-liver disease is the finding of iron deposition and neonatal hemochromatosis (NH). [10, 12] Most cases of NALF and NH are due to gestational alloimmunity, hence the more recent gestational alloimmune liver disease (GALD) has been introduced. [24–26] There seems to be an overlap between the two entities yet their pathogenesis and the autoantibodies are distinct with GALD involving maternal antibodies directed specifically at fetal hepatic antigens resulting in activation of terminal compliment cascade, in utero liver fibrosis, and early onset NALF that is usually isolated to the liver, whereas the autoantibodies (SSA and SSB) in NLE-liver disease are non-liver specific and the disease is not necessarily confined to the liver. [3, 24–26] In a retrospect, some NALF cases with or without a significant cardiac history may in fact have been NLE-liver disease misdiagnosed as GALD considering that screening for anti SSA/SSB is not routinely done in all NALF cases. We had observed a remarkably elevated ferritin in our patient that we had attributed to NLE-liver disease based on the aforementioned reports of neonatal hemochromatosis in association with anti SSA/SSB antibodies. [10, 12] Had the cardiac lesion been absent, we would have treated our patient as a GALD case.

SSA/SSB autoantibodies are highly specific to NLE and their presence is needed to make the diagnosis. [1] Interestingly, while they are likely of maternal origin, only 1% of SSA/SSB positive mothers will have an offspring with NLE. [1, 3] This is because it appears that titers may play a role or that the disease may in fact be multifactorial. [3, 27] While SMA is not 100% specific to AIH-1 and has been described in other liver diseases including non-alcoholic fatty liver disease, it has never been described in association with NLE to our knowledge. [19, 28, 29] SMA are typically of IgG subtype and thus can theoretically cross the placenta just like SSA/SSB, however, only 20–60% of NLE mothers have a history of an autoimmune disorder, mainly lupus, which can rarely be associated with AIH-1. [5, 30] Thus the finding from our case deserves to be highlighted. Nevertheless, we believe that SMA presence in our patient was due to a false-positive reaction rather than a true early onset AIH-1, especially given the patient’s complete resolution of liver injury while being off-immunosuppression. Furthermore, the presence of ANA in our patient, which is not required for NLE diagnosis nor is specific to lupus, may have been maternally acquired along with SSA/SSB. [8]

NLE-liver disease resolves spontaneously in most cases, especially when they present with isolated elevated aminotransferases. [5–9] Some reports showed a satisfactory response to steroids at a dose of 2 mg / kg for 2 weeks. [6, 7, 13] This short duration is highly suggestive of the transient nature of NLE- liver disease in comparison to the cardiac-NLE that is usually more permanent. [1, 3, 20] NLE-liver disease that presents with NALF and NH-like picture should -at least theoretically- respond to GALD management strategies including intravenous immunoglobulins (IVIG) and plasmapheresis or exchange transfusions, but in a recent report of NALF due to NLE, 3 doses of IVIG and 2 exchange transfusions did not reverse the course, and the patient had to be referred for liver transplant, while the other reported case had a fatal outcome. [10, 12] In contrast, our case showed a dramatic response to steroids and continued to have normal levels even 8 weeks after discontinuation of steroids (Table 1).

Recurrence rate of NLE- liver disease in subsequent pregnancies is unknown, however the United States NLE national registry described a recurrent case of NH due to NLE-liver disease in a sibling that died at the age of 6 days. [4] Recurrence rate for cardiac-NLE seems to be much higher at 17–25%. [3]

Prevention trials for cardiac NLE with steroid therapy during pregnancy along with IVIG have been attempted. [3, 31, 32] But such attempts could prove problematic to recommend for routine use given the low prevalence of the disease in SSA/SSB positive otherwise healthy mothers. Mothers who are given a diagnosis of an autoimmune condition, specifically lupus, may decrease their likelihood of passing SSA/SSB autoantibodies when their disease is well controlled with immunosuppression, specifically hydroxychloroquine. [3, 33] In conclusion, NLE-liver disease is a rare cause of neonatal cholestasis and neonatal hemochromatosis that is not typically screened for in that setting. It can resolve spontaneously, or with a short course of prednisolone even in the setting of neonatal hemochromatosis provided there is no or mild synthetic function impairment. Smooth muscle antibodies can be falsely detected in NLE-liver disease.

**Table 1:** Showing the most recent liver parameters at the time of initiation of steroid therapy (Pre-steroids), 4 weeks on steroids (week 4), and 2 weeks after steroids were completely tapered off

	Pre-steroids	week 4	2 weeks post-steroids
TP g\L	58	58	56
ALB g\L	39.9	42.2	40.6
ALP U\L	399	354	274
AST U\L	211	56	39
ALT U\L	160	63	38
GGT U\L	315	126	57
TBIL umol\L	166 (9.7 mg\dl)	18 (1 mg\dl)	12 (0.7 mg\dl)
DBIL umol\L	154 (9 mg\dl)	10 (0.5 mg\dl)	8 (0.4 mg\dl)

*TP* Total protein, *ALB* Albumin, *ALP* Alkaline phosphatase, *AST* Aspartate aminotransferase, *ALT* Alanine aminotransferase, *GGT* Gamma glutamyl transpeptidase, *TBIL* Total bilirubin, *DBIL* Direct bilirubin

## Data Availability

not applicable.

## Abbreviations

NLE: Neonatal lupus erythematosus
NALF: Neonatal acute liver failure
SSA/anti-Ro: Sjogren Syndrome type A antibodies
SSB/anti-La: Sjogren Syndrome type B antibodies
NH: Neonatal hemochromatosis
ANA: Anti-nuclear antibodies
SMA: Smooth muscle antibodies
AIH-1: Autoimmune hepatitis type 1
ASCA: Anti-Saccharomyces Cerevisiae antibodies
ALT: Alanine aminotransferase
AST: Aspartate aminotransferase
ALP: Alkaline phosphatase
GGT: Gamma-glutamyl transferase
GALD: Gestational alloimmune liver disease
SM-ENA: Smith extractable nuclear antigen
IVIG: Intravenous immunoglobulins
